# LLM-based cell type annotation harmonization across single-cell studies using GCTHarmony

**DOI:** 10.21203/rs.3.rs-7151095/v1

**Published:** 2025-08-12

**Authors:** Xingyuan Zhang, Zhicheng Ji

**Affiliations:** 1 Department of Biostatistics and Bioinformatics, Duke University School of Medicine, Durham, NC, USA.; 2 Computational Biology and Bioinformatics Program, Duke University School of Medicine, Durham, NC, USA.

## Abstract

A major challenge in integrating previously analyzed single-cell RNA-seq studies is the inconsistency of cell type annotations. To address this, we developed GCTHarmony, an LLM-based method for harmonizing cell type annotations across single-cell studies. Utilizing OpenAI’s text embedding model, GCTHarmony accurately maps arbitrary cell type annotations to standardized cell ontology terms and reconciles discrepancies in annotation hierarchies across studies. In a real data example, we show that GCTHarmony substantially improves the consistency of cell type annotations across single-cell studies.

Single-cell RNA sequencing (scRNA-seq) technologies have evolved dramatically over the past decade. The emergence of commercial platforms such as 10x Genomics Chromium^1^ has enabled the capture of tens of thousands of individual cells in a single run at low cost. In recent years, a massive number of scRNA-seq studies have been published, often investigating similar tissues or biological questions^2–5^. For example, large consortia such as HuBMAP^3^, Tabula Sapiens^4^, and SenNet^5^ have all generated scRNA-seq data covering normal human tissues in major organs such as the liver, lung, heart, and pancreas. Integrating knowledge across these studies could greatly enhance statistical power and lead to more generalizable discoveries, transforming the fragmented patchwork of results from isolated single-cell studies into cumulative knowledge.

To effectively analyze scRNA-seq data from different studies, one must first identify a set of cell type annotations shared across studies before examining differences in cell type proportions and cell-type-specific gene expression profiles. A common approach is to integrate gene expression matrices across all studies and perform cell clustering and annotation on the integrated dataset^6, 7^. However, this method is time-consuming, requires additional manual effort, and may become computationally infeasible when large datasets are involved. An alternative is to leverage existing cell type annotations from published studies, which avoids extra manual effort and is more computationally efficient. However, substantial discrepancies in cell type annotations across studies often make them not directly comparable. These discrepancies arise primarily from two sources. First, different studies may use varying terms to describe the same cell type, such as “T-cells” versus “T cell”. While humans can easily recognize and resolve such inconsistencies, automated resolution requires complex natural language processing. Second, studies may differ in the hierarchical granularity of their annotations, with some distinguishing T cell subtypes such as naïve and effector T cells, and others grouping them together. Addressing this issue requires mapping annotations to a unified cell type ontology.

Large language models (LLMs), such as GPT-4 developed by OpenAI^8^, have been shown to excel in cell type annotation tasks^9–11^. When provided with a list of marker genes in the prompt message, LLMs can accurately infer corresponding cell types in scRNA-seq studies. However, directly querying LLMs via prompt messages is not an optimal strategy for mapping arbitrary cell type names to standardized cell ontology (CL) terms, for two primary reasons. First, LLMs may produce plausible but incorrect or non-standard terms that do not exist in the official ontology, a phenomenon known as AI hallucination. Second, the number of candidate ontology terms is substantial, and including all of them in a single prompt typically exceeds the context window limitations of most LLMs, making exhaustive and accurate comparison impractical.

Motivated by work such as GenePT^12^, we explore an alternative approach that leverages OpenAI’s text embedding model, which transforms arbitrary text inputs into numerical embedding vectors. The semantic similarity between two texts is then quantified by the distance between their corresponding vectors. To assess the suitability of this embedding model for cell type annotation, we visualized the embedding vectors of 424 commonly referenced cell types cataloged in the Human Reference Atlas (Methods) using Uniform Manifold Approximation and Projection (UMAP) for dimensionality reduction (Figure 1a). The results show that cell type names within the same broad category cluster closely together, while those from different categories are clearly separated. Furthermore, within major categories such as leukocytes, neural cells, epithelial cells, and endothelial cells, cell type names belonging to the same sub-category also form distinct sub-clusters (Figure 1b–e). These findings suggest that the embedding space encodes biologically meaningful relationships among cell type names and supports its potential utility for mapping arbitrary input names to standardized CL terms.

Based on this finding, we developed GCTHarmony, an LLM-based method for harmonizing cell type annotations across single-cell studies (Methods). GCTHarmony has two modules. The first module maps an arbitrary cell type name to its corresponding CL term (Figure 2a). For example, “T-cells” is mapped to the CL term “T cell” (CL:0000084). GCTHarmony utilizes OpenAI’s text embedding model to convert both the arbitrary cell type name and each CL term into numerical embeddings. Cosine similarity is then computed between the embedding of the arbitrary name and each CL term. The CL term with the highest cosine similarity is assigned to the arbitrary cell type name. After identifying CL terms in the first module, GCTHarmony resolves hierarchical discrepancies in the second module (Figure 2b) by mapping cell subtypes (e.g., naive T cell) to their broader parent types (e.g., T cell) based on the ontology tree.

We first evaluated the accuracy of GCTHarmony’s first module, which maps cell type names to CL terms, using two distinct strategies. The first strategy (one-step) directly converts the user-provided cell type names and the commonly used CL terms into embedding vectors using the text embedding model. The second strategy (two-step) first queries the GPT-4o model to generate one-sentence descriptions for both the user-provided cell type names and the commonly used CL terms, and then converts these descriptions into embedding vectors using the text embedding model. Using benchmark datasets and following the evaluation procedure of our previous study^9^, we compared the CL terms generated by both strategies to manually annotated CL terms, treated as the gold standard, across a variety of tissue types and datasets (Figure 2c). The CL terms generated by both strategies show high concordance with the gold standard annotations, with the two-step and one-step strategies achieving full agreement in 74% and 73% of all cases, respectively. The improved performance of the two-step strategy is likely due to the additional semantic enrichment provided by GPT-4o, which allows the text embedding model to better capture the intended meaning of abbreviated or ambiguous terms. Figure 2d presents example cell type names where the one-step strategy failed but the two-step strategy produced correct matches. These examples involve semantically complex names (e.g., those with parentheses) or abbreviations (e.g., “AT1”), which cannot be effectively handled by the embedding model alone. By default, GCTHarmony uses the two-step strategy due to its superior performance, although the one-step strategy is also provided as a simpler alternative.

We next evaluated the full GCTHarmony method for harmonizing cell type annotations across different single-cell studies. We collected five pairs of scRNA-seq datasets (Methods), with each pair derived from the same tissue type (e.g., blood) but generated and analyzed by different research groups. Since each pair was derived from the same tissue, their cell type compositions are expected to be consistent. We compared the correlation of cell type proportions between the two studies in each pair using the annotations downloaded from the original studies, CL terms generated by GCTHarmony’s first module, and CL terms produced by the full GCTHarmony pipeline. For cell types present in only one study, we assigned a proportion of zero in the other study. Figures 2e–g show example correlations for the blood tissue, and Figure 2h summarizes the correlations across all five dataset pairs. Using the original annotations, the correlations were often negative, primarily due to inconsistent naming. This inconsistency was largely resolved after mapping cell type names to CL terms using GCTHarmony’s first module, which substantially improved the correlations. The second module further enhanced consistency by addressing hierarchical discrepancies, resulting in the highest overall correlations. These results suggest that GCTHarmony can robustly harmonize cell type annotations across diverse tissue types.

Similar to GPTCelltype^9^, the first module of GCTHarmony involves the use of the OpenAI API, which is not free of charge. However, the cost is often negligible in real applications. For example, processing 100 cell types costs only around 4 US cents (Figure 2i) for full GCTHarmony method. GCTHarmony is also computationally efficient, requiring only about 100 seconds to process 100 cell types (Figure 2j). Its results are highly reproducible, with agreement scores showing strong consistency across individual runs. (Figure 2k). These results suggest that GCTHarmony is a cost-effective and reproducible approach in practice.

In summary, we developed GCTHarmony, an LLM-based method that accurately maps cell type names to CL terms and harmonizes cell type annotations across single-cell studies. We showed that GCTHarmony improves the consistency of cell type proportions in real data examples. GCTHarmony will streamline the integration of multiple single-cell studies processed and analyzed by different research groups.

## Methods

### Obtaining Cell Ontology (CL) terms

Cell Ontology (CL) terms were downloaded from the Open Biological and Biomedical Ontologies (OBO) Foundry on January 25, 2025 (http://purl.obolibrary.org/obo/cl.owl). The Python package owlready2^13^ was used to manipulate the OWL ontologies. A total of 3076 CL terms were obtained.

Commonly used CL terms were obtained from the ASCT+B Tables available through the Human Reference Atlas portal (https://humanatlas.io/asctb-tables)^14^. In each table, CL terms are listed under the column “CT/1/LABEL.” By concatenating all CL terms across anatomical structures and removing duplicates, a total of 424 unique CL terms were obtained.

### Visualizing CL terms

**Figure 1.**
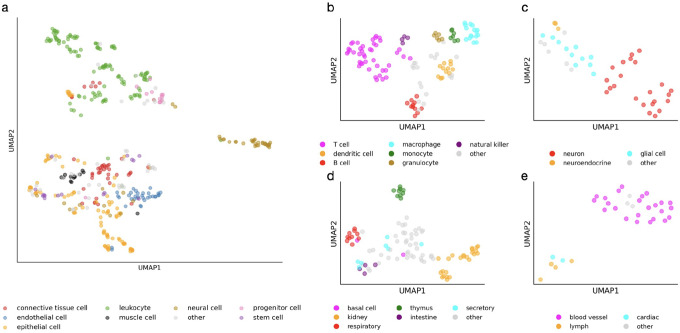
UMAP visualization of numerical embeddings of 424 commonly referenced cell types in the Human Reference Atlas (**a**), cell types belonging to immune cells (**b**), cell types belonging to neural cells (**c**), cell types belonging to epithelial cells (d), and cell types belonging to endothelial cells (**e**).

Each commonly used CL term was converted into an embedding vector using OpenAI’s text-embedding-3-large model. We manually annotated the broad cell type category for each CL term. The embedding vectors of all commonly used CL terms, or of CL terms belonging to a specific broad category, were concatenated into an embedding matrix. The Python package umap^15^ was applied to the embedding matrix using default settings, and the resulting UMAP dimensions were subsequently visualized in Figure 1.

### GCTHarmony

#### First module: mapping cell type names to CL terms

GCTHarmony provides two strategies for mapping cell type names to CL terms. In the one-step strategy, the user-provided cell type name is directly converted into a numerical embedding vector using OpenAI’s text-embedding-3-large model. In the two-step strategy, the gpt-4o-2024–08-06 model is first queried via the OpenAI API with the prompt: “Please use 1 sentence to describe cell type: INPUT-CELL-TYPE”, where INPUT-CELL-TYPE is the user-provided cell type name. The response from GPT-4o is then converted into a numerical embedding vector using the text-embedding-3-large model. The same procedure was used to obtain numerical embeddings of all CL terms in both strategies. The two-step strategy is used by default.

Denote ***z*** as the embedding of the user-provided cell type name and ***v***_*i*_ as the embedding of the *i*th CL term. The user-provided cell type name is then mapped to the *j*th CL term with the highest cosine similarity. Specifically:

$$j=\text{arg}\;\underset{v_{i}\in 𝒱}{\text{max}}\frac{z\cdot v_{i}}{\left\|z\right\|\left\|v_{i}\right\|}$$

Here, 𝒱 is the set of all CL terms being considered. By default, 𝒱 includes only the commonly used CL terms, as many CL terms are rarely used. Users also have the option to set 𝒱 to include all CL terms.

#### Second module: reconciling hierarchical discrepancies

After applying the first module of GCTHarmony to individual studies and obtaining the corresponding CL term lists, GCTHarmony constructs a union set of CL terms across all studies. It then traverses the ontology tree from the root downward. During this traversal, for each node in the union set, GCTHarmony assigns the node’s name to all of its descendant CL terms within the union set.

### Collection of paired scRNA-seq datasets for benchmarking

Five pairs of scRNA-seq datasets used for benchmarking were all downloaded from publicly available sources. The two blood datasets were obtained from^16^ and^17^. The two heart datasets were obtained from^18^ and^19^. The two hippocampus datasets were obtained from^20^ and^21^. The two intestine datasets were obtained from^22^ and^23^. The two kidney datasets were obtained from^24^ and^25^.

Cell type annotations provided by the original studies were directly obtained from their published data.

### Financial cost

The total financial cost of using GCTHarmony is calculated based on the API pricing of OpenAI’s text-embedding-3-large model and the gpt-4o-2024–08-06 model. Specifically, the cost of the text-embedding-3-large model is $0.00013 per 1,000 tokens. The cost of gpt-4o-2024–08-06 model is $0.0025 per 1,000 input tokens and $0.01 per 1,000 output tokens.

### Computational time

To evaluate the computational efficiency of GCTHarmony, we benchmarked its runtime on a machine with an Intel(R) Xeon(R) CPU E5–2699 v4 @ 2.20GHz processor and 64 GB of RAM.

### Reproducibility analysis

To benchmark the reproducibility of GCTHarmony, we applied it to three example tissue types, repeating the process four times. For each tissue, an agreement score was calculated between the gold standard and each GCTHarmony run.

## Figures and Tables

**Figure 2.**
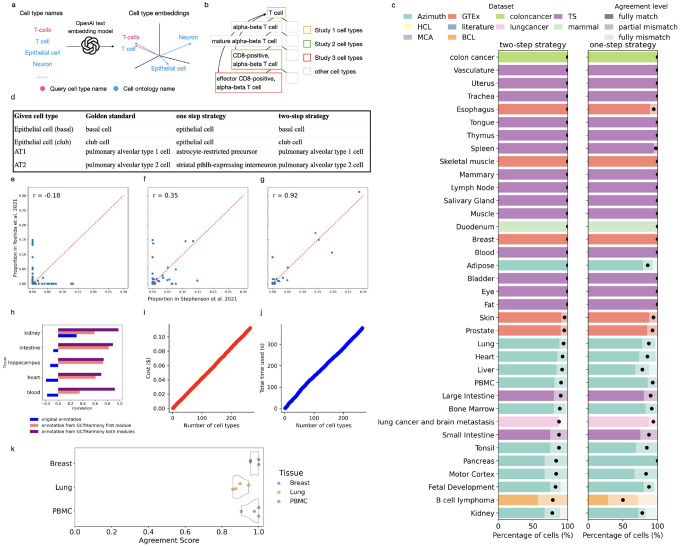
**a–b**, Cartoons demonstrating the first (**a**) and second (**b**) modules of GCTHarmony. **c**, Proportion of cell types with varying agreement levels in each study and tissue for the two strategies. **d**, Four example cell type names, their gold standard CL terms, and the CL terms assigned by the two strategies. **e–g**, Scatter plots comparing cell type proportions in two human blood scRNA-seq studies using the original annotations (**e**), annotations from the first module of GCTHarmony (**f**), and annotations from both modules of GCTHarmony (**g**). The correlation of cell type proportions is shown in the top left corner of each plot. **h**, Correlation of cell type proportions based on the original annotations, annotations from the first module of GCTHarmony, and annotations from both modules of GCTHarmony across different tissue types. **i–j**, Financial cost (**i**) and computational time (**j**) of processing different numbers of cell types using GCTHarmony. **k**, Reproducibility of GCTHarmony. Each data point represents the agreement score from a single GCTHarmony run.

## Data Availability

The GCTHarmony package is is an open source Python software that could be found at the Github repository (https://github.com/Xingyuan-Zhang/GCTHarmony).
